# Risk factors for cut-outs in geriatric intertrochanteric fractures with cephalomedullary nailing after obtaining acceptable reduction: a case–control study

**DOI:** 10.1186/s12891-022-05296-8

**Published:** 2022-04-12

**Authors:** Jian-wen Huang, Xiao-sheng Gao, Yun-fa Yang

**Affiliations:** Department of Orthopaedic Surgery, Guangzhou First People’s Hospital, the Second Affiliated Hospital, School of Medicine, South China University of Technology, Guangzhou, Guangdong 510180 People’s Republic of China

**Keywords:** Cut-out, Intertrochanteric Fractures, Reductions, Cephalomedullary nailing, The elderly

## Abstract

**Background:**

It is irresponsible if we disregard reduction quality to talk about cut-outs in intertrochanteric fractures (ITF) with internal fixation. The aim of this study is to analyze the risk-factors for cut-outs in geriatric ITF with cephalomedullary nailing after obtaining acceptable reduction.

**Methods:**

In order to investigate the risk-factors for cut-outs in geriatric ITF after obtaining acceptable reduction, we retrospectively reviewed 367 patients who underwent cephalomedullary nail for ITF in our department between September 2016 and December 2021. Potential variables including demographic data and radiological parameters (namely the fracture type, Singh index, lateral wall fracture, cephalic nail position, Parker’s ratio index, tip-apex-distance (TAD), and calcar-referenced TAD (CalTAD)) were collected. Logistic regression analysis was performed to identify the significant risk factors for cut-outs.

**Results:**

One hundred twenty-one patients were suitable for this study. Of the 121 cases, nine cases (7.4%) were observed with cut-out or pending cut-out. We found that Age (adjusted odds ratio (OR) 1.158, 95% confidence interval (CI) 1.016 to 1.318, *p* = 0.028), lateral wall fracture (adjusted OR 11.07, 95%CI 1.790 to 68.380, *p* = 0.01), and CalTAD (adjusted OR 1.277, 95%CI 1.005 to 1.622, *p* = 0.045) were independent risk-factors for cut-outs.

**Conclusions:**

Age, lateral wall fracture and CalTAD are independent risk-factors for cut-outs in geriatric ITF with cephalomedullary nailing after obtaining acceptable reduction. In order to avoid cut-outs, an optimal CalTAD is necessary even obtaining acceptable reduction, especially in the over-aged patients with lateral wall fracture.

## Introduction

With the aging population in the global world, hip fractures, which lead to considerable morbidity, mortality and financial burden to family and society, are on a rising trend every year with an expected incidence of 4.5 million in 2050, and at least half of hip fractures are intertrochanteric fractures (ITF) [1].

ITF are generally treated with internal fixation especially cephalomedullary nailing. Although the fixation devices and operative techniques have been developed, cut-outs of cephalic nails, with an incidence rate varying from 3.2% to 20.5% [2–4], are challenging for orthopedists.

We know that cut-outs are usually caused by various factors including bone quality, severity of fracture, reduction, type of fixation, and placement of fixation [5–10]. Unfortunately, what we can change are just reduction, type of fixation, and placement of fixation. We should do our best to achieve good reduction, select suitable fixation and put the fixation in right place. However, letting reduction alone to talk about fixation is meaningless. Therefore, reduction is the first step in cephalomedullary nailing for ITF.

**Fig. 1:**
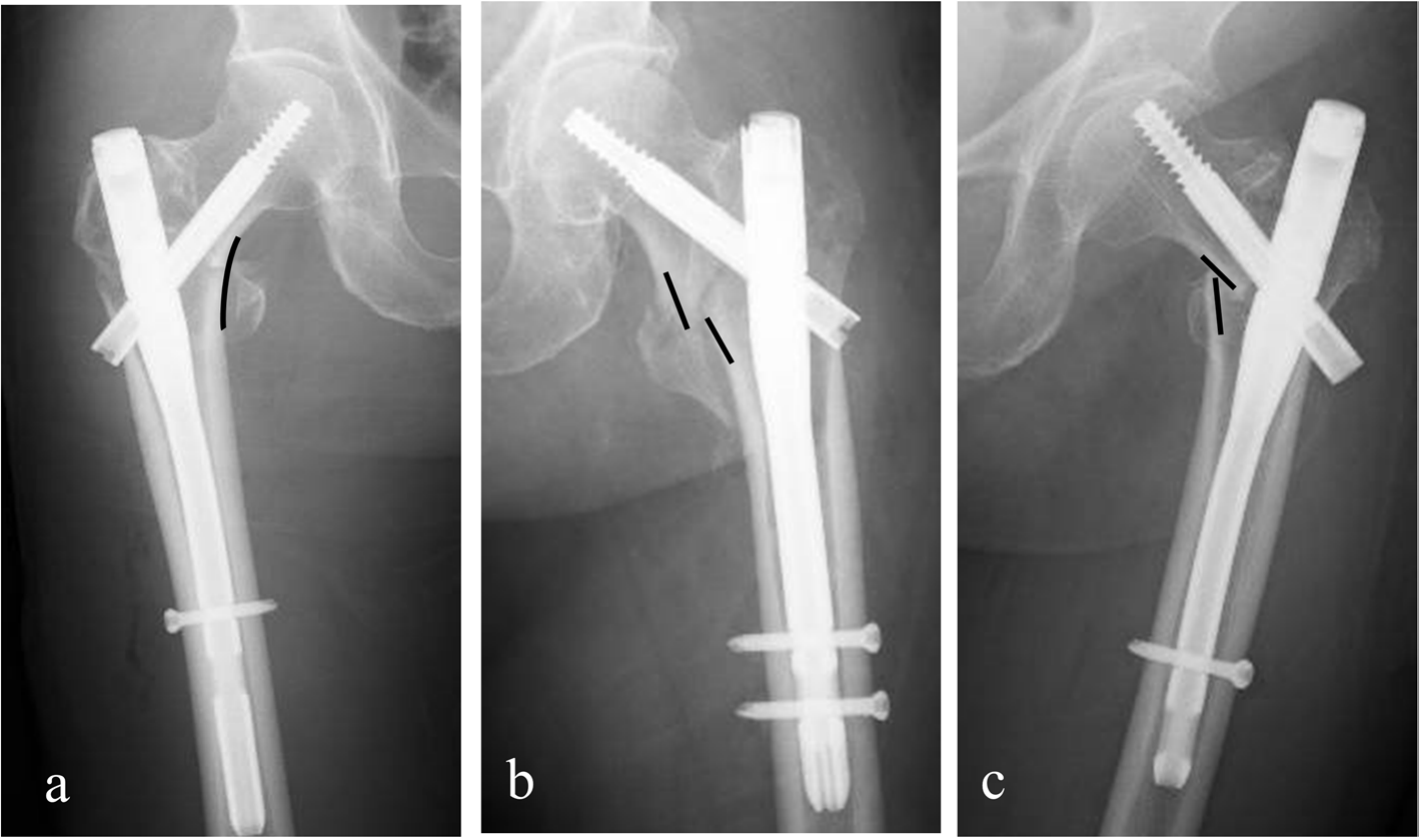
Three types of medial wall reduction. Anatomical reduction (**a**); Positive medial cortex support (PMCS) (**b**); Negative medial cortex support (NMCS) (**c**)

It is a consensus that medial cortex reduction is important to the occurrence of cut-outs [6, 11–14]. If medial cortex reduction is poor, the implant will likely be failed. So far, there are only three types of medial cortex reductions (Fig. 1) included anatomical reduction (Fig. 1a), positive medial cortex support (PMCS) (Fig. 1b) and negative medial cortex support reduction (NMCS) (Fig. 1c), according to the bone contact between the head-neck fragment and femoral shaft in ITF [6, 11]. Anatomical reduction is complete cortex-to-cortex contact between the head–neck fragment and femoral shaft, which is the gold standard of reduction. PMCS or NMCS, defined as the head–neck fragment is displaced medially or laterally to the upper medial edge of the shaft fragment. Actually, since anatomical reduction and PMCS reduction can result in better biomechanical effects and clinical outcomes than NMCS [6, 11], we consider these two types of reductions as the acceptable reduction.

We are concerning why cut-outs cannot be avoided after obtaining acceptable reduction. However, there has been little discussion about the risk-factors for cut-outs after obtaining anatomical reduction or PMCS in published literature. Thus, we hypothesize that there are still some independent risk-factors contributing to this phenomenon. If knowing the details, we may potentially decrease or even avoid cut-outs in geriatric ITF patients with cephalomedullary nailing. Therefore, the purpose of this study is to identify the risk-factors for cut-outs in geriatric ITF with cephalomedullary nailing after obtaining acceptable reduction.

## Methods

### Patients

**Fig. 2:**
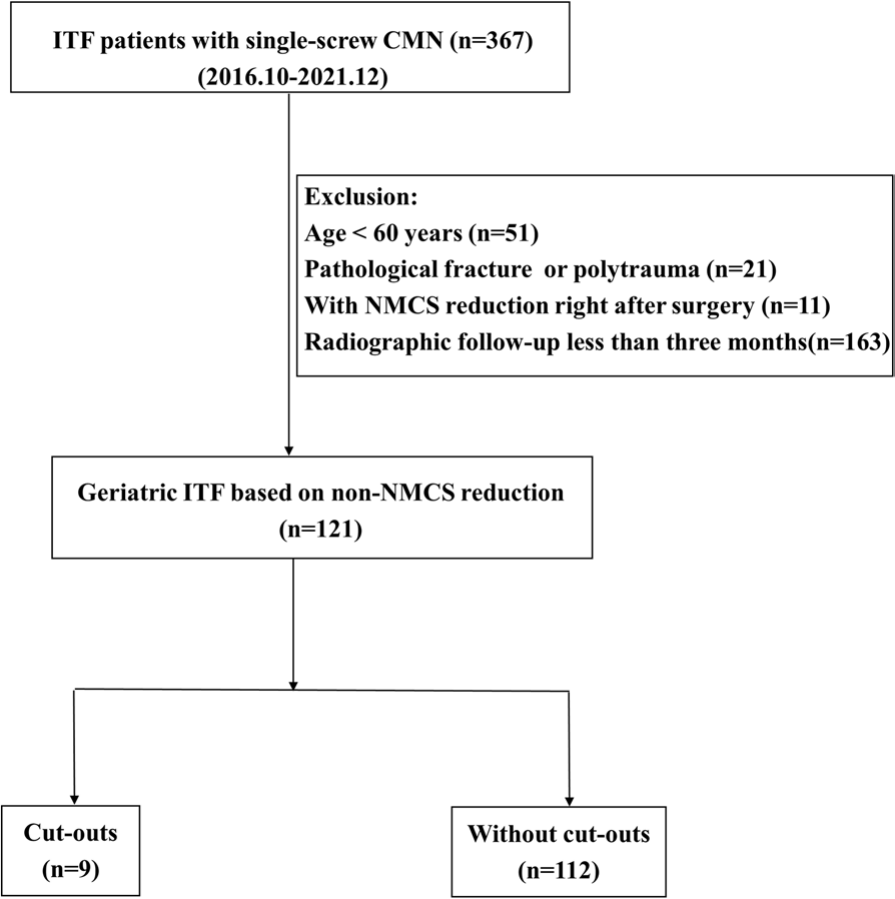
Patients’ flow chart

This study was approved by the Ethics Committee of the authors’ affiliated institution. Clinical and radiological data were collected from the patient record. Inclusion criteria of the present study were: 1) diagnosed as femoral intertrochanteric fractures, 2) underwent cephalomedullary nailing fixation surgery, 3) be hospitalized between September 2016 and December 2021. Exclusion criteria were: 1) age < 60, 2) pathological fracture or polytrauma, 3) a NMCS reduction in the radiographic review right after surgery, 4) a follow-up less than three months. Finally, 121 patients were enrolled in this study (Fig. 2).

### Materials and measurements

Demographic data (including age, gender, fracture site, anesthesia, American Society of Anesthesiologists (ASA) classification and fixation type), and radiological data (namely fracture type, Singh index, lateral wall fracture, cephalic nail position, tip-apex-distance (TAD), calcar-referenced TAD (CalTAD)) were reviewed.

**Fig. 3:**
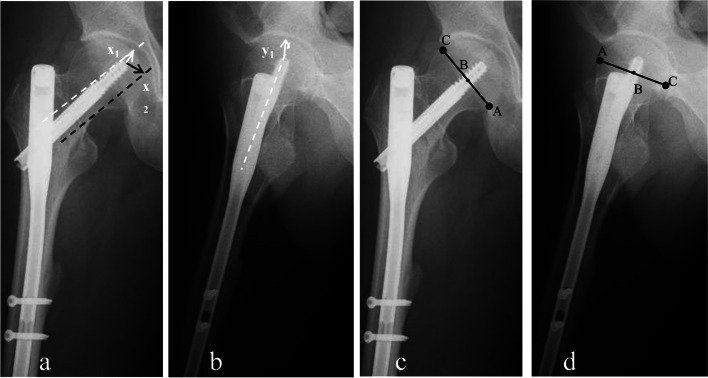
Measurements of radiological parameters. Measurements of TAD and CalTAD were shown on X-ray anteroposterior (AP) view (**a**) and lateral view (**b**) (TAD = $x1+y1,$ CalTAD = $x2+y{1}$). Parker’s ratio index was calculated on AP view (**c**) and lateral view (**d**) (Parker’s ratio = $AB\left/ AC\right.\times 100\%$)

Radiological parameters were obtained from preoperative, intraoperative and the first postoperative X-ray. The fractures were categorized into three groups based on the Arbeitsge-meinschaft für Osteosynthesefragen / Orthopaedic Trauma Association (AO/OTA) classification (2018 version) [15]. Singh Index was used to evaluate osteoporotic degree on preoperative anteroposterior (AP) view [16]. TAD was defined as the sum of distance in millimeters measuring from the tip of cephalic nail to the apex of the femoral head on both AP (Fig. 3a) and lateral radiographs (Fig. 3b) [8]. CalTAD used the same measurement as the TAD in the lateral view (Fig. 3b) but differed in AP view. The measurement of CalTAD in the AP view was moving the apex of femoral head to be adjacent to the medial cortex of the femoral head (Fig. 3a) [9]. The position of cephalic nail was estimated based on the nine zones first reported by Cleveland [10]. Any nail in superior and anterior (namely zone 1) was poor, one either in superior or anterior (namely zone 2, 3, 4 and 7) was questionable, and the other places (namely 5, 6, 8, and 9) were acceptable. Another assessment of nail placement was Parker’s ratio index on AP (Fig. 3c) and lateral views (Fig. 3d) [7].

All calibrations were performed using Digimizer (version 5.4.4 MedCalc Software) by referencing cephalic nails, which both were 10.5 mm in diameter. And all parameters were measured by two independent observers (Jian-wen Huang and Xiao-sheng Gao).

**Fig. 4:**
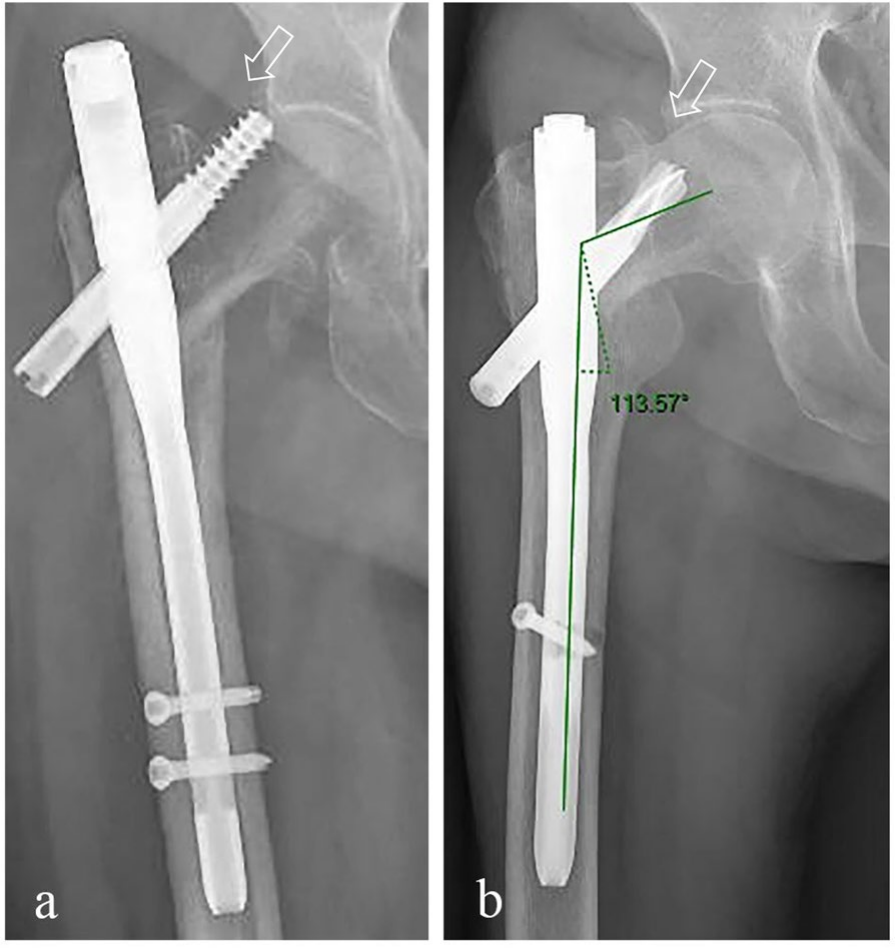
Implant failure types. Implant failure types were shown as the white arrows. Cut-out (**a**), pending cut—out (**b**)

**Fig. 5:**
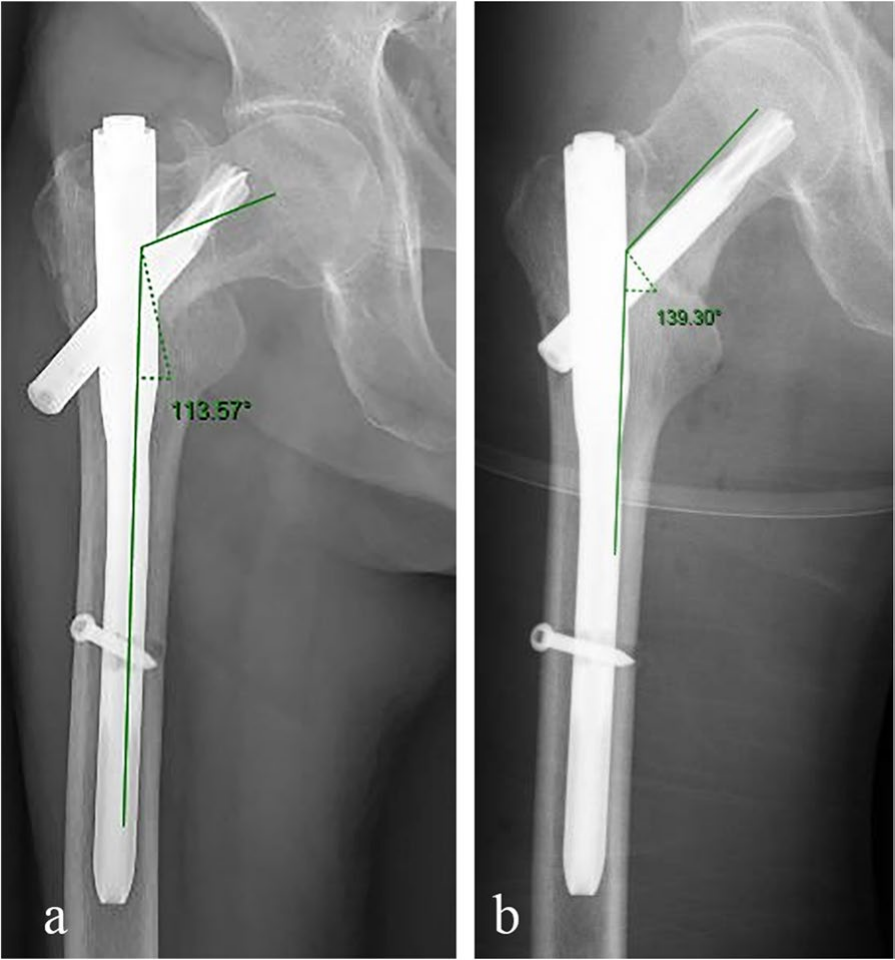
An example of pending cut-out. The neck-shaft-angle (NSA) was measured as 113.57° in the last radiograph on AP view (**a**), which had a more than 20° loss but no penetration or cut-out while comparing with the NSA right after the cephalomedullary nailing surgery (**b**)

Cut-outs were defined as the cut-out that already happened, (Fig. 4a) and pending cut-out (Fig. 4b). Cut-out was meant to the extrusion of cephalic nail from the superior of femoral head. Pending cut-out was determined as the presence of over 20° decrease of neck-shaft angle (NSA) but no penetration or cut-out on AP view in the last radiographic follow-up (Fig. 5a) comparing with the NSA on AP view in the first radiograph right after surgery (Fig. 5b).

#### Statistical analysis

With occurrence of cut-outs as the dependent variable, Student’s t-test or Mann–Whitney U test was used for the continuous variables and Chi-square test for the categorical variables, respectively. A univariate logistics regression was used for crude odds ratio (OR) with 95% confidence interval (CI). The potential significant variables in univariate analysis (*p* < 0.1) were entered into multivariate logistic regression analysis for controlling confounding variables. The receiver operating characteristic (ROC) curve was performed to assess the discrimination ability of significant continuous variable in multivariate logistic regression.

All measuring variables were analyzed for the inter-intra observer reliability with intraclass correlation coefficients (ICCs) for continuous variables and with κ coefficients for categorical variables, respectively. A two-way random effects model with 95% CIs was performed to obtain the average measures ICC. κ coefficients were calculated with 95% CI.

All analyses were performed using SPSS (IBM SPSS Statistic for Windows, Version 25.0. Armonk, NY: IBM Corp). All tests were two-sided and the *p*-value below 0.05 was considered statistically significant.

## Results

A total of 121 ITF patients were included for full analysis. Of these 121 patients, nine cases (7.4%) were observed with cut-outs (including six cases of cut-out and three of pending cut-outs). 112 patients (87.3%) were observed with radiological union without cut-outs at the last follow-up. Median [interquartile range, IQR] follow-up time was 6 [3, 14.5] months. The results of reliability analysis between two independent observers for measuring parameter were shown in (Table 1) according to the rating by Landis [17].

**Table 1:** Reliability between two independent observers for measuring variables

Variable	ICC or κ	95% CI	Reliability
Singh index	0.561	0.430 to 0.692	Moderate
Cleveland zone	0.730	0.634 to 0.826	Excellent
AO/OTA classification	0.738	0.620 to 0.856	Excellent
Parker ratio (AP)	0.916	0.882 to 0.941	Almost perfect
Parker ratio (Lat)	0.924	0.893 to 0.947	Almost perfect
Tip-apex distance	0.906	0.868 to 0.933	Almost perfect
Calcar reference tip-apex distance	0.939	0.914 to 0.957	Almost perfect

*AO/OTA* AO Foundation and Orthopaedic Trauma Association, *AP* anteroposterior view, *LAT* lateral view, *ICC* intraclass correlation coefficient, *CI* confidence interval

In the univariate analysis of demographic data (Table 2), there were no significant differences in gender, fracture site, anesthesia and ASA score between the two groups except the age at the fracture. The patients with cut-outs had significantly higher age than those without cut-outs (median age [IQR], 86.0 [82.0, 89.5] *vs.* 81.0 [73.5, 85.0], *p* = 0.043).

**Table 2:** Univariate analysis of demographic data

Factor	Overall (*n* = 121)	Without cut-outs (*n* = 112)	Cut-outs (*n* = 9)	*p*-value	Crude OR (95% CI)
Age, years	82.0 [75.0, 86.0]	81.0 [73.5, 85.0]	86.0 [82.0, 89.5]	0.043^a^	1..09 (0.99 to 1.20)
Gender (%)				0.310^**b**^	2.26 (0.45 to 11.41)
Male	46 (38.0)	44 (39.3)	2 (22.2)		
Female	75 (62.0)	68 (60.7)	7 (77.8)		
Fracture site (%)				0.460^**b**^	1.67 (0.43 to 6.54)
Left	68 (56.2)	64 (57.1)	4 (44.4)		
Right	53 (43.8)	48 (42.9)	5 (55.6)		
Anesthesia (%)				0.899^**b**^	0.90 (0.18 to 4.59)
Spinal	92 (76.0)	85 (75.9)	7 (77.8)		
General	29 (24.0)	27 (24.1)	2 (22.2)		
ASA (%)				0.313^**b**^	N/A
2	53 (43.8)	51 (45.5)	2 (22.2)		
3	65 (53.7)	58 (51.8)	7 (77.8)		
4	3 (2.5)	3 (2.7)	0 (0)		

^a^ Mann–Whitney U test, the results are shown as median [interquartile range]

^b^ Chi-square test, the results are shown as number (percentage)

*N/A* not applicable

*OR* odds ratio, *CI* confidence interval, *ASA* American Society of Anesthesiologists

In the radiological data analysis (Table 3), significant differences were not found in AO/OTA fracture classification, Singh index, fixation type, Parker’s ratio on AP and lateral views. 14 patients (11.6%) were observed with lateral wall fractures before surgery, and no iatrogenic fractures were found in the postoperative X-ray review. Four cases in 9 of the cut-outs group had lateral wall fracture preoperatively (4/9, 44.4%) while only 10 cases in 102 of the patients without cut-outs had lateral wall fracture before surgery (10/102, 8.9%), the difference was significant (*p* < 0.001). Also, the cut-outs group had significant higher mean TAD (22.44 ± 5.76 mm, *vs.* 18.93 ± 4.90 mm, *p* = 0.025) and mean CalTAD (27.99 ± 5.89 mm *vs.* 22.07 ± 5.68 mm, *p* = 0.003). In terms of cephalic nail placement, the cut-outs group had lower rate of acceptable placement (6/9, 66.7% *vs.* 100/112, 89.3%), but the difference was not significant (*p* = 0.072).

**Table 3:** Univariate analysis of radiological factors

Factor	Overall (*n* = 121)	Without cut-outs (*n* = 112)	Cut-outs (*n* = 9)	*p*-value	OR (95% CI)
AO/OTA (%)				0.305^**b**^	N/A
31A1	64 (52.9)	61 (54.5)	3 (33.3)		
31A2	52 (43.0)	46 (41.1)	6 (66.7)		
31A3	5 (4.1)	5 (4.5)	0 (0)		
Singh index (%)				0.544^**b**^	1.56 (0.37 to 6.54)
≤ 3	69 (57.0)	63 (56.3)	6 (66.7)		
> 3	52 (43.0)	49 (43.7)	3 (33.3)		
Fixation type (%)				0.941^b^	0.95 (0.22 to 4.01)
Helical blade	39 (32.2)	36 (32.1)	3 (33.3)		
Lag screw	82 (67.8)	76 (67.9)	6 (66.7)		
Lateral wall fracture (%)				0.001^b^	8.16 (1.88 to 35.36)
No	107 (88.4)	102 (91.1)	5 (55.6)		
Yes	14 (11.6)	10 (8.9)	4 (44.4)		
Nail position quality (%)				0.072^b^	4.14 (0.84 to 20.36)
Poor	2 (1.7)	2 (1.8)	0		
Questionable	13 (10.7)	10 (8.9)	3 (33.3)		
Acceptable	106 (87.6)	100 (89.3)	6 (66.7)		
Parker’s ratio (AP)	49.04 ± 8.44	48.49 ± 7.95	54.54 ± 12.44	0.193	1.09 (0.89 to 1.18)
Parker’s ratio (Lat.)	49.32 ± 9.23	49.17 ± 8.52	51.16 ± 16.38	0.538	1.02 (0.95 to 1.11)
TAD, mm	18.93 ± 4.90	18.65 ± 4.74	22.44 ± 5.76	0.025	1.16 (1.01 to 1.33)
CalTAD, mm	22.51 ± 5.88	22.07 ± 5.68	27.99 ± 5.89	0.003^a^	1.19 (1.05 to 1.35)

^a^ Student’s t-test, the results are shown as mean ± standard deviation

^b^ Chi-square test, the results are shown as number (percentage)

*OR* odds ratio, *CI* confidence interval, *AO/OTA* AO Foundation and Orthopaedic Trauma Association, *AP* anteroposterior view, *Lat*. lateral view, *TAD* tip-apex-distance, *CalTAD* calcar referenced tip-apex-distance

In the multivariate analysis (Table 4), age, lateral wall fracture, cephalic nail placement, TAD and CalTAD were selected for confounders controlling. Statistical differences were found in age (Adjusted OR 1.158, 95%CI 1.016 to 1.318, *p* = 0.028), lateral wall fracture (Adjusted OR 11.070, 95%CI 1.790 to 68.380, *p* = 0.01) and CalTAD (Adjusted OR 1.277, 95%CI 1.005 to 1.622, *p* = 0.045) at multivariate analysis.

**Table 4:** The results of multivariate logistic regression analysis

Factor	*p*-value	Adjusted OR	95% CI
Age	0.028	1.158	1.016 to 1.318
Lateral wall fracture	0.010	11.070	1.790 to 68.380
Cleveland zone 1 to 4, 7	0.564	1.848	0.229 to 14.909
TAD	0.713	1.046	0.825 to 1.326
CalTAD	0.045	1.277	1.005 to 1.622

*OR* odds ratio, *CI* confidence interval, *TAD* tip-apex-distance, *CalTAD* calcar-referenced tip-apex-distance

**Fig. 6:**
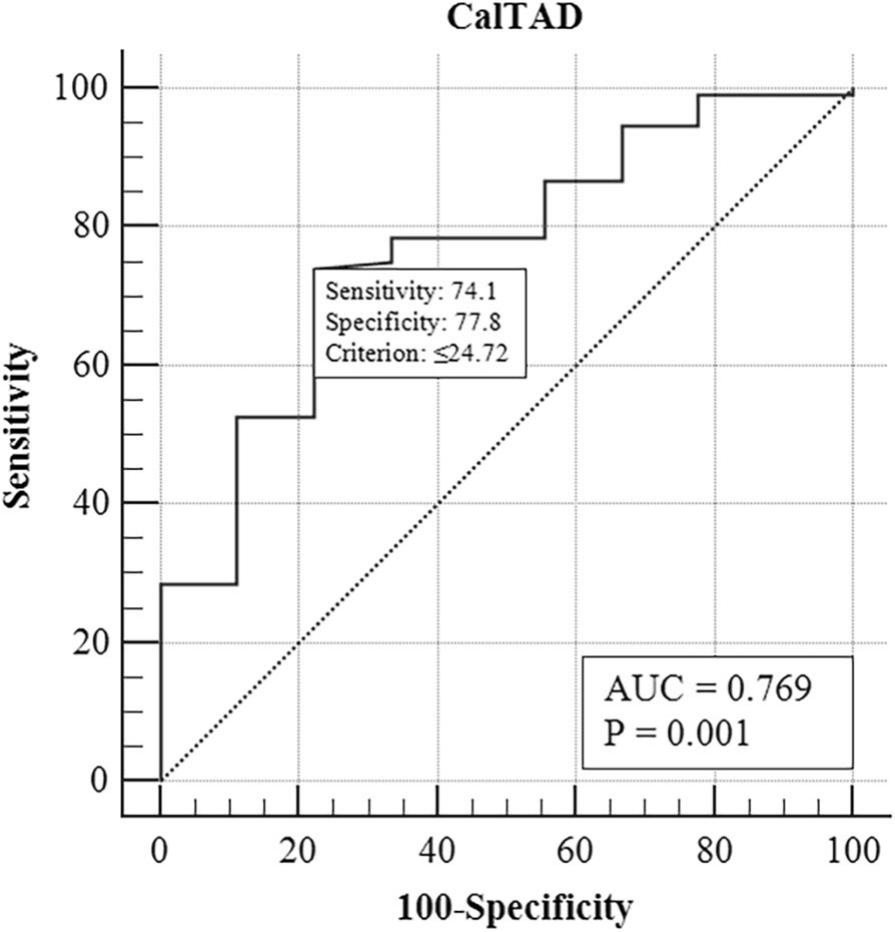
The Receiver Operating Characteristic (ROC) curve analysis. The Receiver Operating Characteristic (ROC) curve shown the best threshold for CalTAD in preventing cut-outs was 24.72 mm (the area under the curve (AUC) = 0.769, Sensitivity = 74.1, Specificity 77.8, *p* = 0.001).

The ROC curve analysis was performed to determine predictive effect of CalTAD (Fig. 6). After application of Youden test which balanced the highest values of sensitivity and specificity, the results indicated that a best cut-off value for CalTAD was 24.72 mm.

## Discussion

We know that letting reduction alone to talk about fixation is meaningless and anatomical reduction is the best for fracture fixation. However, anatomical reduction is very difficult for all the geriatric ITF patients because of their poor general condition caused by aging. Therefore, acceptable reduction is necessary to the geriatric ITF patients [6, 11]. Actually, acceptable reduction in ITF patients is not enough because cut-outs still happen even in the cases with acceptable reduction. In this retrospective study, we find that age, lateral wall fracture and CalTAD are independent risk-factors for cut-outs in geriatric ITF with cephalomedullary nailing after obtaining acceptable reduction.

As we known, the bone quality is on a downtrend with age increasing [18, 19]_,_ which may increase the risk of cut-outs. Age was a reliable predictor for cut-outs both at univariate analysis (*p* = 0.043) and multivariate analysis (*p* = 0.028) in our study. The patients with cut-outs, with a median age of 86 years, is significantly higher than those without cut-outs. We consider that rather than the reduction quality and the position of internal fixation, bone quality is primary for cut-outs in patients over 85 years. As for the evaluation of bone quality on X-ray, Singh index also was not observed with difference (*p* = 0.544). Klatte [20] concluded that the Singh index did not correlate with bone mineral density. Some studies [9, 21] also found that Singh index was not associated with cut-outs, with which we hold the same viewpoint.

The presence of lateral wall fracture had been identified as a prognostic factor for cut-outs [22–24]. It is reported that the greater trochanter fracture was associated with poor functional outcome [25]. Our study further confirms the lateral wall fracture is an independently significant risk-factor for cut-outs at the univariate analysis (*p* = 0.001) and multivariate analysis (Adjust OR 11.070, 95% CI 1.790 to 68.380, *p* = 0.01). The risk of cut-outs is 11-fold higher in the patients with lateral wall fracture than those with lateral wall integrity. Theoretically, the cephalomedullary nail can be the partial replacement of lateral wall. But the rotation stability and lateral buttress for the proximal fragment will be lost in the existence of lateral wall fracture. Gotfried [22] reported that the lateral wall integrity was also important in the cases which the trochanteric portion was not fully broken. Basically, in all the ITF in our study we obtained acceptable reduction intraoperatively. When the medial wall cortical contact is relatively optimal, as the corresponding part, the lateral wall integrity comes into a leading role because anatomical mechanical balance can be mostly recovered from the two wall bony supports.

CalTAD is more reasonable than TAD to predict cut-outs of ITF fixation. Actually, TAD, a reliable predictor for screw cut-outs first reported by Baumgaertner [8], was widespread used for placement of cephalic nail at the time of operation, which hold that TAD should be lower than 25 mm for preventing cut-out in both extramedullary and intramedullary nailings. In terms of cephalomedullary nailing, John [21] reported that TAD with 23.56 mm was the most sensitive for predicting cut-out. Our study confirmed a bigger TAD was the risk-factor for cut-out (Crude OR 1.16, 95% CI 1.01 to 1.33, *p* = 0.025) by univariate analysis, but not as an independent predictor (Adjusted OR 1.046, 95% CI 1.005 to 1.622, *p* = 0.713) by multivariate analysis. The CalTAD, as the counterpart of TAD, favored an inferior-central region of femoral head as the optimal cephalic nail position. CalTAD was concluded as the only significant risk-factor for cut-outs in cephalomedullary nailing by Kashigar [9]. Coinciding with Kashigar’s conclusion, our study further confirms the CalTAD is associated with cut-outs both at univariate analysis (*p* = 0.003) and multivariate analysis (OR 1.277, 95% CI 1.005 to 1.622, *p* = 0.045). There is consensus in the literature [26–28] that individual differences and geometrical characteristics are correlated to the precise cut-off value, which is the reason for the slight difference between our study and Kashigar’s. Some studies [28, 29] recommended that TAD was more reliable than CalTAD for cut-outs. The discrepancy between our study and theirs may be partial explained, as suggested in a biomechanical study by Kane [30], lower central placement with TAD higher than 25 mm provided equal if not superior stability to central – central placement with TAD lower than 25 mm. We postulate that a lower CalTAD is likely offset the risk of cut-outs produced by higher TAD. Moreover, Kuzyk [31] suggested that inferior lag screw position produced the highest axial and torsional stiffness, particularly in acceptable bone contact, which favored CalTAD as an optimal parameter for screw position.

There are several limitations in our study. First, this is a retrospective study which is not only susceptible to bias, but also fails to obtain some significant parameters such as body mass index, bone mineral density and postoperative weight-bearing status. Second, we only enrolled patients who underwent single-screw cephalomedullary nailing, hence the conclusion is likely not applied to other internal fixations. Third, there is a small size patients enrolled in this study. Thus, biomechanical experiments and prospective multicentre studies are needed to make further investigation.

## Conclusions

Age, lateral wall fracture and CalTAD are independent risk-factors for cut-outs in geriatric ITF with cephalomedullary nailing after obtaining acceptable reduction. In order to avoid cut-outs, an optimal CalTAD is necessary even obtaining acceptable reduction, especially in the over-aged patients with lateral wall fracture.

## Data Availability

The dataset supporting the conclusions of this manuscript is available upon request by contacting the corresponding author.

## Abbreviations

ITF: Intertrochanteric fractures
PMCS: Positive medial cortex support
NMCS: Negative medial cortex support
ASA: American Society of Anesthesiologists
TAD: Tip-apex distance
CalTAD: Calcar-referenced tip-apex distance
AO/OTA: Arbeitsge-meinschaft für Osteosynthesefragen/Orthopaedic Trauma Association
AP: Anteroposterior
NSA: Neck-shaft angle
OR: Odds ratio
CI: Confidence interval
IQR: Interquartile range
ROC: Receiver operating characteristic curve
